# Supplementary material for: A Potent PDK4 Inhibitor for Treatment of Heart Failure with Reduced Ejection Fraction
Source: Cells. 2023 Dec 30;13(1):87. doi: 10.3390/cells13010087 (PMC10777911; doi:10.3390/cells13010087)
Supplement: Supplementary file 1 [file cells-13-00087-s001.zip › cells-2748420-supplementary.pdf]

A Potent PDK4 Inhibitor for Treatment of Heart Failure with Reduced Ejection Fraction

Supplementary methods

Unless otherwise noted, reagents and solvents were commercially available and used without further purification. All dry solvents such as MeOH and THF were purchased from Kanto Chemical Co., Inc. For thin layer chromatography (TLC) analysis, precoated silica gel plates with a fluorescent indicator (Merck 60 F254) were used. Flash chromatography was carried out with Kanto Chemical silica gel (Kanto Chemical, silica gel 60N, spherical neutral, 0.040-0.050 mm) or Merck silica gel 230-400 mesh ASTM (60N, 0.040-0.063 mm). Optical rotations were measured on a JASCO P-1010 polarimeter. Melting point (m.p.) were measured on a SRS MPA-100 optimelt. Infrared (IR) spectra were recorded on a Horiba FT-210 spectrometer. NMR spectra were measured on a JEOL JNM-ECA-500 spectrometer with ^{1}H NMR at 500 MHz and ^{13}C NMR at 125 MHz. Chemical shifts were reported in ppm from the internal solvent peaks for chloroform-*d1* ($CDCl_3$) (^{1}H; δ= 7.26 ppm, ^{13}C; δ= 77.16 ppm), dimethylsulfoxide-*d6* (DMSO-*d6*) (^{1}H; δ= 2.50 ppm, ^{13}C; δ= 39.52 ppm). ^{1}H NMR data were reported as follows: chemical shift (integration, multiplicity (s = singlet, d = doublet, t = triplet, m = multiplet, br = broad), coupling constants (Hz)). The high-resolution mass spectra (HRMS) were performed on a JEOL JMS-700 MStation and JEOL JMS-T100LP.

Experimental procedure

(*E*)-4-(2,5-Dimethoxy-3-methylphenyl)-3-(methoxycarbonyl)but-3-enoic acid (**SI1**)

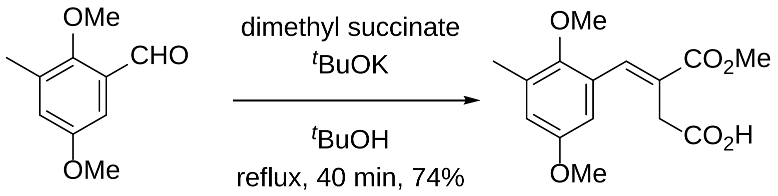

1 **SI1**

According to the method reported by Maloney *et al.*,[1] **1** was prepared from *O*-cresol. To a solution of ^{t}BuOK (2.9 g, 25.8 mmol, 1.2 eq.) in ^{t}BuOH (36.7 mL, 0.6 M) at reflux under N_2 atmosphere was added a solution of **1** (3.87 g, 21.5 mmol) and dimethyl succinate (3.1 mL, 23.7 mmol, 1.1 eq.) in ^{t}BuOH (35.0 mL, 0.6 M) dropwise. After being stirred for 40 min at reflux, the reaction mixture was cooled to room temperature, and concentrated in vacuo to remove ^{t}BuOH. The residue was then acidified by the addition of 2 M HCl aqueous solution (30 mL), and extracted with Et_2O (50 mL x3). The combined organic layer was extracted with 2 M NaOH aqueous solution (40 mL x5), then the combined aqueous layer

was acidified by addition of 4 M HCl aqueous solution (150 mL). The resulted acid layer was extracted with Et_2O (100 mL x3), and dried over Na_2SO_4, filtered and concentrated. The residue was purified with flash column chromatography (silica gel; $CHCl_3$/MeOH = 40/1) to afford **SI1** (4.69 g, 74% yield) as a pale yellow solid.

m.p.: 151.3-151.4 ^{o}C.

IR (diamond prism)νcm^{-1}: 2954, 2927, 2838, 2370, 2356, 1699, 1643, 1602, 1471, 1427, 1373, 1292, 1216, 1178, 1151, 1099, 1060, 1006, 941, 866, 760.

^{1}H NMR (500 MHz, $CDCl_3$)δ(ppm): 7.98 (s, 1H), 6.75 (d, *J* = 2.8 Hz, 1H), 6.67 (d, *J* = 2.8 Hz, 1H), 3.86 (s, 3H), 3.76 (s, 3), 3.62 (s, 3H), 3.54 (s, 2H), 2.28 (s, 3H)..

^{13}C NMR (125 MHz, $CDCl_3$)δ(ppm): 175.1, 168.3, 155.5, 151.1, 139.5, 132.9, 128.7, 126.1, 118.3, 111.9, 61.5, 55.7, 52.7, 34.1, 16.4

HRMS (EI) *m*/*z* 294.1106 [M]+, calcd 294.1103 for $C_{15}H_{18}O_6$.

3-(2,5-Dimethoxy-3-methylbenzyl)-4-methoxy-4-oxobutanoic acid (**2**):

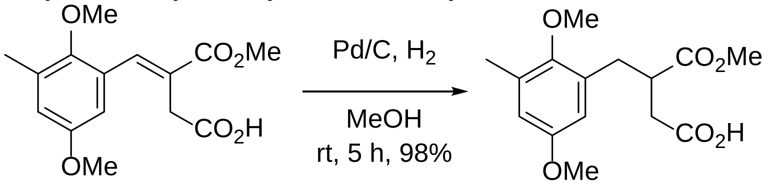

SI1 **2**

To a solution of **SI1** (436.0 mg, 1.48 mmol) in MeOH (15.0 mL, 0.1 M) at room temperature was added 0.5% Pd/C (218.0 mg). After being stirred at room temperature under H_2 atmosphere for 5 h, the reaction mixture was filtered with Celite® to remove Pd/C, and the filtrates were concentrated. The residue was purified with flash column chromatography (silica gel; $CHCl_3$/MeOH = 40/1) to afford **2** (420.0 mg, 96% yield) as a light yellow solid.

IR (diamond prism)νcm^{-1}: 2983, 2958, 2923, 2838, 2738, 2642, 1697, 1600, 1473, 1438, 1324, 1215, 1176, 1099, 1060, 1005, 865, 760.

^{1}H NMR (500 MHz, $CDCl_3$)δ(ppm): 6.60 (d, *J* = 2.9 Hz, 1H), 6.48 (d, *J* = 2.9 Hz, 1H), 3.73 (s, 3H), 3.68 (s, 3H), 3.66 (s, 3H), 3.16 (m, 1H), 3.02 (dd, *J* = 13.8, 5.8 Hz, 1H), 2.77 (dd, *J* = 13.8, 8.9 Hz, 1H), 2.69 (dd, *J* = 17.2, 9.2 Hz, 1H), 2.45 (dd, *J* = 17.2, 4.6 Hz, 1H), 2.21 (s, 3H).

^{13}C NMR (125 MHz, $CDCl_3$)δ(ppm): 176.8, 174.9, 155.6, 151.1, 132.4, 131.8, 115.3, 113.3, 60.7, 55.6, 52.2, 41.9, 34.6, 32.4, 16.6.

HRMS (EI) *m*/*z* 296.1259 [M]+, calcd 296.1260 for $C_{15}H_{20}O_6$.

Methyl 5,8-dimethoxy-7-methyl-4-oxo-1,2,3,4-tetrahydronaphthyl-2-carboxylate (**3**):

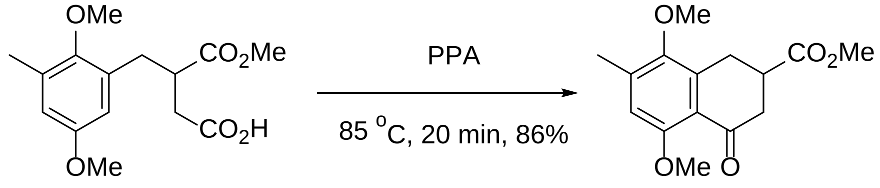

2 **3**

To a solution of **2** (1.81 g, 6.11 mL) in polyphosphoric acid (30.5 mL, 0.2 M) was stirred at 85 °C for 20 min, then the reaction mixture was cooled to room temperature. The mixture was quenched by pouring into ice water (50 mL) with vigorous stirring, and extracted with $CHCl_3$ (50 mL x3). The combined organic layer was dried over Na_2SO_4, filtered and concentrated. The residue was purified with column chromatography (silica gel; Hex/EtOAc = 40/1) to afford **3** (1.46 g, 86% yield) as a colorless solid.

m.p.: 165.1-166.3 °C.

IR (diamond prism)νcm^{-1}:1728, 1699, 1683, 1621, 1440, 1321, 1257, 1234, 1166, 1132, 1056, 1003, 902, 854, 835, 794, 678, 628.

^{1}H NMR (500 MHz, $CDCl_3$)δ(ppm): 6.67 (s, 3H), 3.86 (s, 3H), 3.71 (s, 3H), 3.69 (s, 3H), 3.36 (ddd, *J* = 16.0, 3.5, 1.7 Hz, 1H), 3.04 (m, 1H), 2.97 (dd, *J* = 16.0, 10.3 Hz, 1H), 2.85 (ddd, *J* = 17.2, 4.6, 1.7 Hz, 1H), 2.77 (dd, *J* = 17.2, 10.9 Hz, 1H), 2.34 (s, 3H).

^{13}C NMR (125 MHz, $CDCl_3$)δ(ppm): 194.7, 173.9, 156.7, 149.1, 138.9, 136.7, 120.2, 112.8, 60.4, 56.3, 52.3, 42.3, 39.5, 26.5, 17.2.

HRMS (EI) *m/z* 278.1153 [M]+, calcd 278.1154 for $C_{15}H_{18}O_5$.

Methyl 5,8-dimethoxy-7-methyl-1,2,3,4-tetrahydronaphthalene-2-carboxylate (**4**):

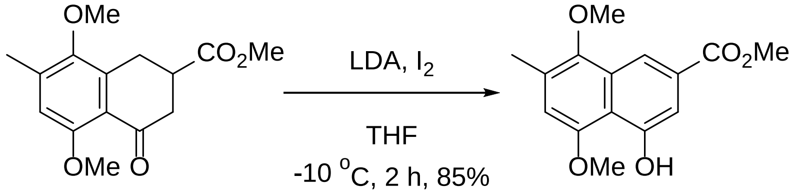

3 **4**

To a solution of **3** (1.4 g, 5.03 mmol) in trimethylsilane (2.5 mL, 15.7 mmol, 3.1 eq.) was added TFA (7.6 mL, 0.7 M) at room temperature. After being stirred for 5 min, the reaction mixture was concentrated. The residue was purified with flash column chromatography (silica gel; $CHCl_3$/MeOH = 100/1) to afford **4** (1.3 g, 98% yield) as a colorless solid.

m.p.: 73.1-73.4 °C.

IR (diamond prism)νcm^{-1}: 3001, 2948, 2906, 2885, 2840, 1722, 1602, 1583, 1481, 1432, 1403, 1360, 1336, 1307, 1234, 1188, 1088, 1056, 1018, 984, 960, 839, 817, 775, 746.

^{1}H NMR (500 MHz, $CDCl_3$)δ(ppm): 6.68 (s, 1H), 3.87 (s, 3H), 3.72 (s, 3H), 3.70 (s,

3H), 3.17 (dd, *J* = 16.1, 4.0 Hz, 1H), 2.89 (ddd, *J* = 17.5, 5.7, 3.2 Hz, 1H), 2.81 (dd, *J* = 16.1, 10.9 Hz, 1H), 2.65 (m, 1H), 2.55 (ddd, *J* = 17.5, 11.5, 6.3 Hz, 1H), 2.30 (s, 3H), 2.22. (m, 1H), 1.75 (ddd, *J* = 24.1, 11.5, 5.7 Hz, 1H).
^{13}C NMR (125 MHz, $CDCl_3$)δ(ppm): 176.4, 153.2, 149.9, 129.5, 127.9, 123.3, 109.7, 60.0, 55.5, 51.9, 39.6, 26.4, 25.4, 22.8, 16.3.
HRMS (EI) *m*/*z* 264.1355 [M]+, calcd 264.1362 for $C_{15}H_{20}O_4$.

<u>Methyl 5,8-dimethoxy-7-methyl-2-naphtoate (**SI2**):</u>

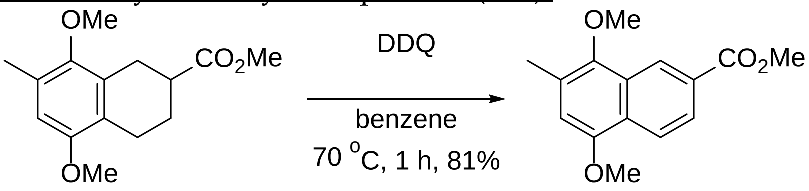

4 **SI2**

To a solution of **4** (141.0 mg, 0.53 mmol) in benzene (2.65 mL, 0.2 M) under N_2 atmosphere was added DDQ (243.0 mg, 1.07 mmol, 2.0 eq.) at room temperature. After being stirred at 70 °C for 1 h, the reaction mixture was cooled to room temperature, then quenched with $NaHCO_3$ aq. solution (2.0 mL). The resulting two layers were separated, and the organic layer was washed with $NaHCO_3$ aq. solution (5 mL x3). The organic layer was dried over Na_2SO_4, filtered and concentrated. The residue was purified with flash column chromatography (silica gel; $CHCl_3$) to afford **SI2** (112.0 mg, 81% yield) as a colorless solid.
m.p.: 106.4-106.9 °C.
IR (diamond prism)νcm^{-1}: 3001, 2948, 2838, 1718, 1633, 1600, 1508, 1394, 1344, 1274, 1245, 1220, 1195, 1159, 1108, 1024, 995, 973, 912, 838, 759, 713.
^{1}H NMR (500 MHz, $CDCl_3$)δ(ppm): 8.76 (d, *J* = 1.7 Hz, 1H), 8.23 (d, *J* = 8.6 Hz, 1H), 8.00 (dd, *J* = 8.6, 1.7 Hz, 1H), 6.71 (s, 1H), 3.98 (s, 3H), 3.97 (s, 3H), 3.89 (s, 3H), 2.46 (s, 3H).
^{13}C NMR (125 MHz, $CDCl_3$)δ(ppm): 167.6, 151.4, 148.0, 128.1 (x2) 127.3, 126.9, 124.8, 124.2, 122.8, 109.4, 61.8, 55.8, 52.4, 16.5.
HRMS (EI) *m*/*z* 260.1056 [M]+, calcd 260.1049 for $C_{15}H_{16}O_4$.

<u>Methyl 7-methyl-5,8-dioxo-5,8-dihydronaphthalene-2-carboxylate (**5**):</u>

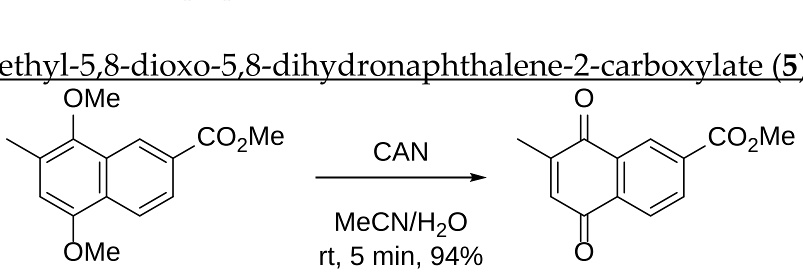

SI2 **5**

To a solution of **SI2** (80 mg, 0.31 mmol) in MeCN/H_2O (15.4 mL, 0.02 M) was added CAN (421.1 mg, 0.77 mmol, 2.5 eq.) at 0 °C. After being stirred for 30 min, the reaction mixture was added H_2O (1 mL), and extracted with EtOAc (1 mL x3). The combined organic layer was dried over Na_2SO_4, filtered and

concentrated. The residue was purified with column chromatography (silica gel; Hex/EtOAc = 5/1) to afford **5** (66.5 mg, 94% yield) as a yellow solid.
m.p.: 133.5-134.2 °C.
IR (diamond prism)νcm^{-1}: 2362, 1728, 1665, 1622, 1603, 1433, 1349, 1286, 1233, 1156, 1117, 1101, 946, 902, 866, 781, 767, 720, 693, 661, 465, 416.
^{1}H NMR (500 MHz, $CDCl_3$)δ(ppm): 8.74 (d, *J* = 1.7 Hz, 1H), 8.37 (dd, J = 8.0, 1.7 Hz, 1H), 8.14 (d, *J* = 8.0 Hz, 1H), 6.9 (q, *J* = 1.7 Hz, 1H), 3.99 (s, 3H), 2.23 (d, *J* = 1.7 Hz, 3H).
^{13}C NMR (125 MHz, $CDCl_3$)δ(ppm): 184.7, 184.3, 165.5, 148.8, 135.8, 134.8, 134.7, 134.2, 132.2, 127.9, 126.4, 52.8, 16.6.
HRMS (EI) *m/z* 230.0573 [M]+, calcd 230.0579 for $C_{15}H_{16}O_5$.

<u>Methyl 4-hydroxy-5,8-dimethoxy-7-methyl-2-nanpthoate (**6**):</u>

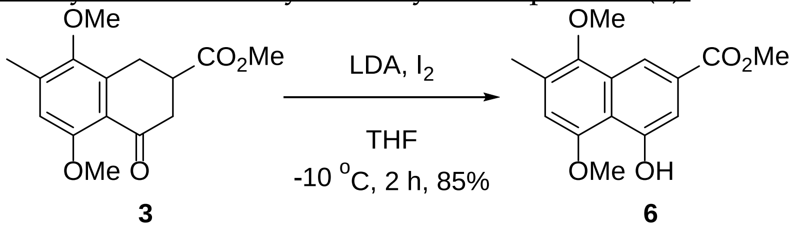

To a solution of diisopropylamine (122.0 μL, 0.87 mmol, 1.2 eq) in THF (7.2 mL, 0.1 M) at -78 °C under N_2 atmosphere was added ^{n}BuLi (1.64 M in Hexane, 524.0 μL, 0.86 mmol, 1.2 eq.) dropwise, and the reaction mixture was stirred at -78 °C for 15 min to prepare a lithium diisopropylamine (LDA) in THF solution, which was warmed to -10 °C. Then the solution of **3** (200.0 mg, 0.72 mmol) in THF (7.2 mL, 0.1 M) was added to the LDA solution. After being stirred for 10 min at the temperature, I_2 (109.0 mg, 0.86 mmol, 1.2 eq.) was added, and stirred for 1 h. The reaction mixture was quenched with $Na_2S_2O_3$ aq., and extracted with $CHCl_3$ (15 mL x 3). The combined organic layer was dried over Na_2SO_4, filtered and concentrated. The residue was purified with flash column chromatography (silica gel; Hex/EtOAc = 40/1) to afford **6** (169.7 mg, 85% yield) as a colorless solid.
m.p.: 133.9-134.2 °C.
IR (diamond prism)νcm^{-1}: 3355, 2935, 1739, 1720, 1616, 1508, 1438, 1380, 1272, 1207, 1164, 1122, 1022, 914, 867, 825, 771, 659.
^{1}H NMR (500 MHz, $CDCl_3$)δ(ppm): 9.36 (s, 1H), 8.26 (d, *J* = 1.7 Hz, 1H), 7.40 (d, *J* = 1.7 Hz, 1H), 6.68 (s, 1H), 4.03 (s, 3H), 3.95 (s, 3H), 3.86 (s, 3H), 2.42 (s, 3H).
^{13}C NMR (125 MHz, $CDCl_3$)δ(ppm): 167.3, 155.2, 151.9, 149.1, 130.4, 129.5, 126.9, 116.7, 115.8, 109.4, 109.3, 61.6, 56.5, 52.4, 16.3.
HRMS (EI) *m/z* 276.1000 [M]+, calcd 276.0998 for $C_{15}H_{16}O_5$.

Methyl 4-acetoxy-5,8-dimethoxy-7-methyl-2-nanpthoate (**SI3**):

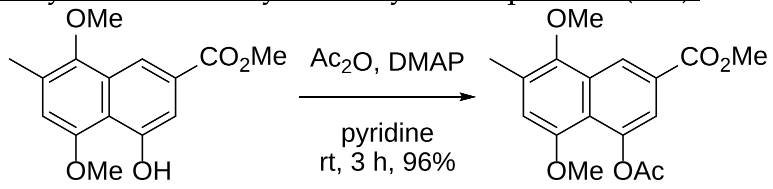

6 SI3

To a solution of **6** (50.0 mg, 0.18 mmol) in pyridine (1.8 mL, 0.1 M) at room temperature under N_2 atmosphere was added Ac_2O (19.0 μL, 0.20 mmol, 1.1 eq.) and DMAP (2.2 mg, 0.018 mmol, 0.1 eq.). After being stirred for 3 h, the reaction mixture was quenched with H_2O, and extracted with $CHCl_3$ (5 mL x3). The combined organic layer was dried over Na_2SO_4, filtered and concentrated. The residue was purified with flash column chromatography (silica gel; Hex/EtOAc = 20/1) to afford **SI3** (54.8 mg, 96% yield) as a colorless solid.
m.p.: 141.6-141.9 ^{o}C.
IR (diamond prism)νcm^{-1}: 2996, 2938, 2842, 1754, 1720, 1608, 1446, 1349, 1276, 1207, 1126, 1022, 971, 906, 836, 763, 655.
^{1}H NMR (500 MHz, $CDCl_3$)δ(ppm): 8.69 (d, *J* = 1.7 Hz, 1H), 7.61 (d, *J* = 1.7 Hz, 1H), 6.75 (s, 1H), 3.96 (s, 3H), 3.90 (s, 3H), 3.87 (s, 3H), 2.43 (s, 3H), 2.37 (s, 3H).
^{13}C NMR (125 MHz, $CDCl_3$)δ(ppm): 170.2, 166.6, 151.0, 148.5, 147.1, 130.6, 128.0, 127.8, 123.3, 120.6, 118.3, 111.9, 61.8, 56.5, 52.5, 21.0, 16.4.
HRMS (EI) *m/z* 318.1113 [M]+, calcd 318.1103 for $C_{17}H_{18}O_6$.

Methyl 4-acetoxy-7-methyl-5,8-dioxo-5,8-dihydronaphthalene-2-carboxylate (**7**):

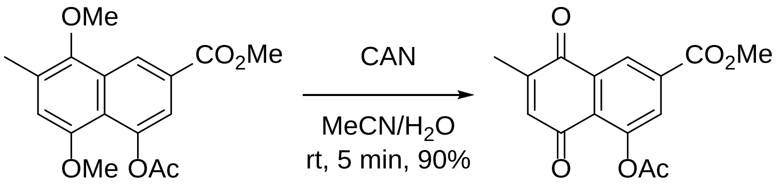

SI3 7

To a solution of CAN (193.0 mg, 0.35 mmol, 2.2 eq.) in H_2O (0.32 mL, 0.5 M) was slowly added to a solution of **SI3** (51.9 mg, 0.16 mmol) in MeCN (0.32 mL) at room temperature. After being stirred for 5 min, the reaction mixture was diluted with H_2O (5 mL) and extracted with EtOAc (3 mL x3). The combined organic layer was dried over Na_2SO_4, filtered and concentrated. The residue was purified with flash column chromatography (silica gel; Hex/EtOAc = 20/1) to afford **7** (41.5 mg, 90% yield) as a pale yellow solid.
m.p.: 135.5-135.8 ^{o}C.
IR (diamond prism)νcm^{-1}: 2962, 2927, 2850, 2410, 2283, 1766, 1716, 1654, 1427, 1357, 1307, 1276, 1238, 1191, 1079, 1033, 933, 867, 813, 767.
^{1}H NMR (500 MHz, $CDCl_3$)δ(ppm): 8.65 (d, *J* = 1.8 Hz, 1H), 7.99 (d, *J* = 1.7 Hz, 1H), 6.75 (d, *J* = 1.7 Hz, 1H), 3.98 (s, 3H), 2.44 (s, 3H), 2.18 (d, *J* = 1.8 Hz, 3H).

^{13}C NMR (125 MHz, $CDCl_3$)δ(ppm): 184.1, 183.1, 169.3, 164.6, 149.6, 147.6, 137.2, 125.8, 134.2, 130.2, 126.3, 126.0, 53.1, 21.1, 16.3.
HRMS (EI) *m/z* 288.0635 [M]+, calcd 288.0634 for $C_{15}H_{12}O_6$.

<u>Methyl 4-hydroxy-7-methyl-5,8-dihydronaphthalene-2-carboxylate (**8**):</u>

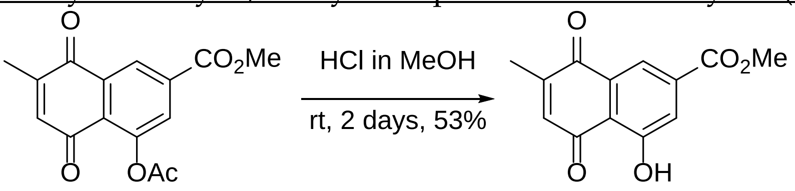

7 **8**

To a solution of **7** (10.0 mg, 0.034 mmol) in MeOH (365 μL, 0.1 M) was added 2 M HCl in MeOH (73 μL) at 0 °C. After being stirred for 48 h at room temperature, the reaction mixture was concentrated. The residue was purified with flash column chromatography (silica gel; Hex/EtOAc = 40/1) to afford **8** (4.4 mg, 53% yield) as a yellow solid.
m.p.: 142.3-142.7 °C.
IR (diamond prism)νcm^{-1}: 3103, 3060, 2960, 2921, 2850, 1722, 1672, 1645, 1614, 1571, 1432, 1342, 1240, 1218, 1118, 1083, 1043, 931, 898, 736, 701, 667, 624.
^1H NMR (500 MHz, $CDCl_3$)δ(ppm): 11.9 (s, 1H), 8.24 (d, *J* = 1.8 Hz, 1H), 7.90 (d, *J* = 1.8 Hz, 1H), 6.86 (dd, *J* = 3.2, 1.7 Hz, 1H), 3.97 (s, 3H), 2.22 (d, *J* = 1.7 Hz, 3H).
^{13}C NMR (125 MHz, $CDCl_3$)δ(ppm): 190.2, 184.1, 165.2, 161.2, 150.4, 137.1, 135.7, 132.4, 125.2, 119.8, 117.5, 53.0, 16.8.
HRMS (EI) *m/z* 246.0526 [M]+, calcd 246.0528 for $C_{13}H_{10}O_5$.

<u>5,8-Dimethoxy-6-methyl-2-naphthoic acid (**10**):</u>

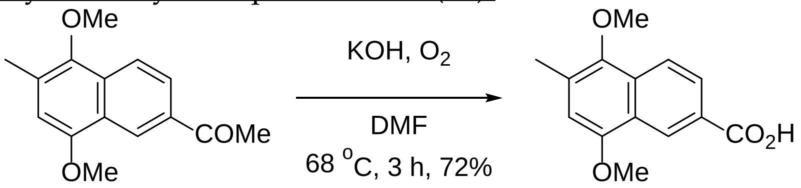

9 **10**

According to the method reported by Nyland *et al.[2]* and Joshi *et al.[3]*, **7** was prepared from Vitamin K_3. To a solution of **9** (200.0 mg, 0.82 mmol) in DMF (20 mL, 0.04 M) under O_2 atmosphere was added powdered KOH (260.0 mg, 4.60 mmol, 5.6 eq.) at room temperature. After being stirred at 68 °C for 3 h, the reaction mixture was poured into cooled water (20 mL). the reaction mixture was washed with Et_2O (20 mL x3), and the resulting aqueous layer was acidified by the addition of 4 M HCl aq. Ssolution (10 mL). The mixture was then extracted with $CHCl_3$ (20 mL x3). The combined organic layer was dried over Na_2SO_4, filtered and concentrated to afford **10** (145.2 mg, 72% yield) as a pale yellow solid without purification.
m.p.: 192.0-192.1 °C.

IR (diamond prism)νcm^{-1}: 3008, 2938, 2850, 2630, 2557, 1986, 1681, 1623, 1457, 1376, 1292, 1218, 1137, 1083, 1014, 925, 833, 755, 721, 663.
^{1}H NMR (500 MHz, DMSO-d_6)δ(ppm): 8.74 (s, 1H), 8.03 (dd, J = 8.6, 1.8 Hz, 1H), 8.00 (dd, J = 8.6, 1.8 Hz, 1H), 6.91 (s, 1H), 3.98 (s, 3H), 3.79 (s, 3H), 2.50 (s, 3H).
^{13}C NMR (125 MHz, DMSO-d_6)δ(ppm): 167.4, 151.6, 146.0, 129.9, 129.2, 126.5, 126.0, 124.6, 123.4, 121.6, 108.5, 61.0, 55.8, 16.2.
HRMS (EI) m/z 246.0899 [M]+, calcd 246.0892 for $C_{14}H_{14}O_4$.

Methyl 5,8-dimethoxy-6-methyl-2-naphthoate (**SI4**):

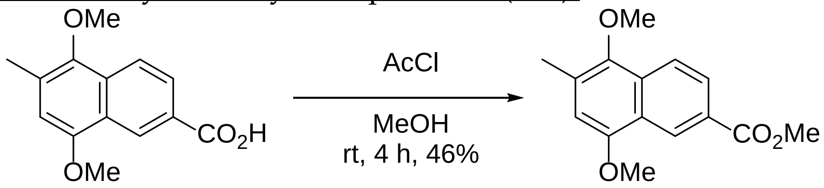

10 **SI4**

To a solution of **10** (12.1 mg, 49.1 μmol) in MeOH (2.0 mL, 0.025 M) was added acetyl chloride (10.5 μL, 147.3 μmol, 3.0 eq.) at room temperature. After being stirred for overnight, the reaction mixture was quenched with H_2O (3.0 mL). The resulted two layers were extracted with $CHCl_3$ (5.0 mL x3), and washed with sat. $NaHCO_3$ aq. (5.0 mL). The combined organic layer was dried over Na_2SO_4, filtered and concentrated. The residue was purified with column chromatography (silica gel; Hex/EtOAc = 4/1) to afford **SI4** (5.8 mg, 46% yield) as a white solid.
m.p.: 93.5-94.3 ^{o}C.
IR (diamond prism)νcm^{-1}: 2931, 2848, 2360, 2341, 1627, 1600, 1462, 1437, 1391, 1378, 1357, 1346, 1234, 1003, 929, 866, 782, 721, 680, 664, 518, 487, 433, 408.
^{1}H NMR (500 MHz, $CDCl_3$)δ(ppm): 8.95 (d, J = 1.7 Hz, 1H), 8.09 (dd, J = 8.6, 1.7 Hz, 1H), 8.05 (d, J = 8.6 Hz, 1J), 6.65 (s, 1H), 3.99 (s, 3H), 3.97 (s, 3H), 3.86 (s, 3H), 2.47 (s, 3H).
^{13}C NMR (125 MHz, $CDCl_3$)δ(ppm): 167.4, 152.5, 146.9, 130.7 (x2), 129.1, 126.0, 125.6, 124.4, 121.8, 107.5, 61.3, 55.7, 52.1, 16.5.
HRMS (EI) m/z 260.1043 [M]+, calcd 260.1049 for $C_{15}H_{16}O_5$.

Methyl 6-methyl-5,8-dioxo-5,8-dihydronaphthalene-2-carboxylate (**11**):

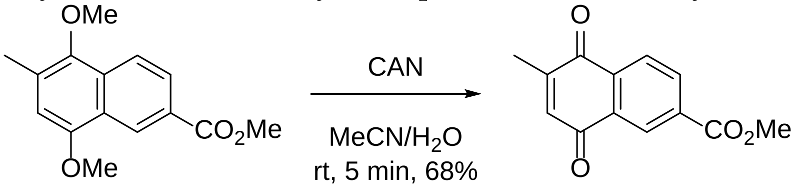

SI4 **11**

To a solution of **SI4** (10.2 mg, 39.2 μmol) in MeCN/H_2O (0.2 mL, 0.02 M) was added CAN (54.8 mg, 100.0 μmol, 2.5 eq.) at 0 ^{o}C. After being stirred for 30

min, the reaction mixture was added H_2O (1 mL), and extracted with EtOAc (1 mL x3). The combined organic layer was dried over Na_2SO_4, filtered and concentrated. The residue was purified with column chromatography (silica gel; Hex/EtOAc = 5/1) to afford **11** (6.1 mg, 68% yield) as a yellow solid.
m.p.: 154.7-155.7 °C.
IR (diamond prism)νcm^{-1}: 1716, 1659, 1625, 1603, 1443, 1377, 1354, 1325, 1224, 1165, 1125, 1075, 974, 876, 785, 768, 689, 670, 645, 486.
^{1}H NMR (500 MHz, $CDCl_3$)δ(ppm): 8.69 (d, *J* = 1.2 Hz, 1H), 8.36 (dd, *J* = 8.0, 1.7 Hz, 1H), 8.17 (d, *J* = 8.0 Hz, 1H), 6.91 (q, *J* = 1.7 Hz, 1H), 3.98 (s, 3H), 2.22 (d, *J* = 1.7 Hz, 3H).
^{13}C NMR (125 MHz, $CDCl_3$)δ(ppm): 185.0, 184.1, 165.5, 148.6, 136.1, 134.9, 134.8, 134.3, 132.4, 127.6, 126.9, 52.8, 16.6.
HRMS (EI) *m*/*z* 230.0571 [M]+, calcd 230.0579 for $C_{13}H_{10}O_4$.

tert-Butyl (5,8-dimethoxy-6-methyl-2-naphthoyl)glycinate (**12a**):

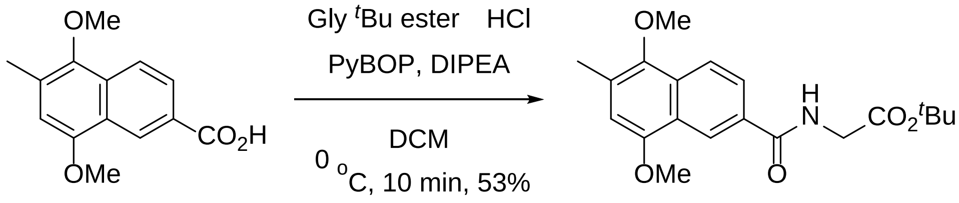

10 **12a**

To a solution of **10** (300.0 mg, 1.22 mmol) in DCM (24.4 mL, 0.05 M) was added glycine *tert*-butyl ester hydrochloride (225.0 mg, 1.34 mmol, 1.1 eq.), DIPEA (0.53 mL, 3.05 mmol, 2.5 eq.) and PyBOP (760.0 mg, 1.46 mmol, 1.2 eq.) at room temperature. After being stirred for 10 min, the reaction mixture was cooled to 0 °C, and quenched with MeOH (1 mL) and H_2O (10 mL). The resulting two layers were separated, and the aqueous layer was extracted with $CHCl_3$ (15 mL x3). The combined organic layer was dried over Na_2SO_4, filtered and concentrated. The residue was purified with flash column chromatography (silica gel; $CHCl_3$/MeOH = 100/1) to afford **12a** (231.6 mg, 53% yield) as a pale yellow solid.
m.p.: 171.1-171.3 °C.
IR (diamond prism)νcm^{-1}: 3355, 3004, 2985, 2935, 2850, 1735, 1535, 1469, 1361, 1307, 1226, 1153, 1103, 987, 917, 844, 794, 755, 671, 624.
^{1}H NMR (500 MHz, $CDCl_3$)δ(ppm): 8.64 (d, *J* = 1.8 Hz, 1H), 8.06 (d, *J* = 8.6 Hz, 1H), 7.95 (dd, *J* = 8.6, 1.8 Hz, 1H), 6.82 (brs, 1H), 6.64 (s, 1H), 4.20 (d, *J* = 5.2 Hz, 2H), 3.98 (s, 3H), 3.85 (s, 3H), 2.46 (s, 3H), 1.52 (s, 9H).
^{13}C NMR (125 MHz, $CDCl_3$)δ(ppm): 169.5, 167.6, 152.3, 147.0, 130.2, 129.9, 128.5, 124.9, 124.5, 122.3, 121.8, 107.7, 82.6, 61.5, 55.7, 42.8, 28.2, 16.6.
HRMS (EI) *m*/*z* 359.1734 [M]+, calcd 359.1733 for $C_{20}H_{25}NO_5$.

tert-Butyl (6-methyl-5,8-dioxo-5,8-dihydronanphthalene-2-carbonyl)glycinate (**SI5a**):

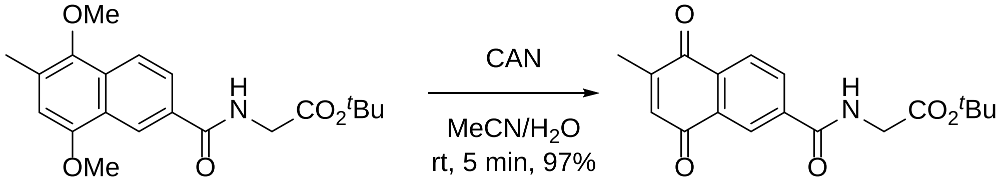

12a **SI5a**

To a solution of CAN (671.1 mg, 1.22 mmol, 2.2 eq.) in H_2O (1.1 mL, 0.5 M) was slowly added to a solution of **12a** (200.0 mg, 0.56 mmol) in MeCN (1.1 mL) at room temperature. After being stirred for 5 min, the reaction mixture was diluted with H_2O (10 mL) and extracted with EtOAc (7 mL x3). The combined organic layer was dried over Na_2SO_4, filtered and concentrated. The residue was purified with flash column chromatography (silica gel; Hex/EtOAc = 40/1) to afford to **SI5a** (176.4 mg, 97% yield) as a pale yellow solid.

m.p.: 148.3-148.7 ^{o}C.

IR (diamond prism)νcm^{-1}: 3351, 2985, 2927, 1739, 1646, 1515, 1434, 1369, 1295, 1226, 1157, 944, 897, 848, 809, 755, 690.

^{1}H NMR (500 MHz, $CDCl_3$)δ(ppm): 8.40 (d, J = 1.5 Hz, 1H), 8.22 (dd, J = 8.0, 2.0 Hz, 1H), 8.17 (d, J = 8.5 Hz, 1H), 6.90 (d, J = 2.0 Hz, 1H), 6.84 (brs, 1H), 4.17 (d, J = 5.5 Hz, 2H), 2.22 (d, J = 1.5 Hz, 3H), 1.51 (s, 9H).

^{13}C NMR (125 MHz, $CDCl_3$)δ(ppm): 185.0, 184.2, 169.0, 165.5, 148.7, 138.6, 136.1, 134.1, 132.7, 132.4, 127.3, 124.3, 83.0, 42.8, 28.2, 16.6.

HRMS (EI) *m/z* 330.1346 [M]+, calcd 330.1341 for $C_{18}H_{20}NO_5$.

(6-Methyl-5,8-dioxo-5,8-dihydronaphthalene-2-carbonyl)glycine (**13a**):

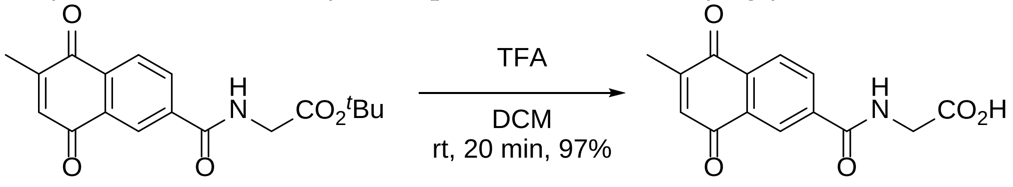

SI5a **13a**

SI5a (100.0 mg, 0.30 mmol) was dissolved in 50% TFA/DCM (30 mL, 0.01 M), and the reaction mixture was stirred at room temperature. After being stirred for 20 min, the reaction mixture was concentrated. The residue was purified with flash column chromatography (silica gel; $CHCl_3$/MeOH = 20/1) to afford **13a** (79.4 mg, 97% yield) as a light brownish yellow solid.

m.p.: 163.0-163.2 ^{o}C.

IR (diamond prism)νcm^{-1}: 3278, 3043, 2923, 2869, 1704, 1654, 1542, 1411, 1334, 1284, 1241, 1164, 1014, 941, 875, 802, 690, 644.

^{1}H NMR (500 MHz, $CDCl_3$)δ(ppm): 8.40 (d, J = 1.7 Hz, 1H), 8.25 (dd, J = 8.6, 1.7 Hz, 1H), 8.20 (d, J = 8.6 Hz, 1H), 6.93 (d, J = 1.7 Hz, 1H), 6.84 (brs, 1H), 4.20 (d, J =

5.2 Hz, 2H), 2.25 (d, *J* = 1.7 Hz, 1H).
^{13}C NMR (125 MHz, $CDCl_3$)δ(ppm): 184.8, 184.3, 171.2, 165.2, 148.6, 138.3, 135.6, 133.7, 132.6, 132.0, 126.7, 124.5, 41.5, 16.2.
HRMS (EI) *m*/*z* 274.0722 [M]+, calcd 274.0715 for $C_{14}H_{12}NO_5$.

<u>*tert*-Butyl *O*-(*tert*-butyl)-*N*-(5,8-dimethoxy-6-methyl-2-nanphthoyl)-L-serinate (**12b**):</u>

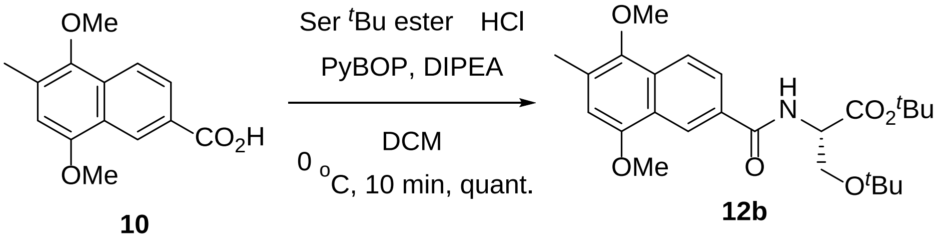

10 **12b**

To a solution of **10** (330.0 mg, 1.22 mmol) in DCM (24.4 mL, 0.05 M) was added *O*-*tert*-butyl-L-serine *tert*-butyl ester hydrochloride (340.0 mg, 1.34 mmol, 1.1 eq.), DIPEA (0.53 mL, 3.05 mmol, 2.5 eq.) and PyBOP (760.0 mg, 1.46 mmol, 1.2 eq.) at room temperature. After being stirred for 10 min, the reaction mixture was cooled to 0 °C, then quenched with the addition of MeOH (1.0 mL) and H_2O (10.0 mL). The resulting two layers were separated, and the aqueous layer was extracted with $CHCl_3$ (15 mL x3). The combined organic layer was dried over Na_2SO_4, filtered and concentrated. The residue was purified with column chromatography (silica gel; Hex/EtOAc = 10/1) to afford **12b** (543.1 mg, quant. Yield) as colorless amorphous.
$[\alpha]_D^{27.0}$: +39.0 ° (*c* = 0.1, $CHCl_3$).
IR (diamond prism)νcm^{-1}: 2973, 2935, 1735, 1654, 1508, 1461, 1361, 1226, 1153, 1087, 1018, 979, 910, 840, 732.
^{1}H NMR (500 MHz, $CDCl_3$)δ(ppm): 8.67 (d, *J* = 1.7 Hz, 1H), 8.07 (d, *J* = 9.2 Hz, 1H), 7.94 (dd, *J* = 8.6, 1.7 Hz, 1H), 7.14 (dd, *J* = 8.6 Hz, 1H), 6.64 (s, 1H), 4.88 (ddd, *J* = 9.2, 8.6, 8.0 Hz, 1H), 3.98 (s, 3H), 3.90 (dd, *J* = 9.2, 2.9 Hz, 1H), 3.85 (s, 3H), 3.71 (dd, *J* = 8.0, 2.9 Hz, 1H), 2.46 (s, 3H), 1.51 (s, 9H), 1.17 (s, 9H).
^{13}C NMR (125 MHz, $CDCl_3$)δ(ppm): 169.9, 167.2, 152.3, 146.9, 130.3, 130.1, 128.3, 124.8, 124.5, 122.2, 122.0, 107.6, 82.0, 73.2, 62.6, 61.4, 55.7, 53.8, 28.2, 27.5, 16.5.
HRMS (EI) *m*/*z* 468.2354 [M+Na]+, calcd 468.2362 for $C_{25}H_{35}NO_6Na$.

<u>*tert*-Butyl *O*-(*tert*-butyl)-*N*-(6-methyl-5,8-dioxo-5,8-dihydronaphthalene-2-carbonyl)-L-serinate (**SI5b**):</u>

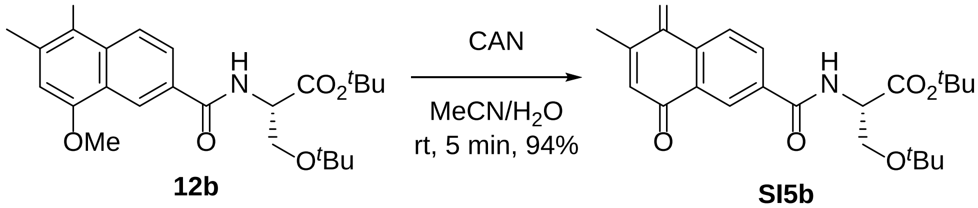

12b **SI5b**

To a solution of CAN (1.34 g, 2.44 mmol, 2.2 eq.) in H_2O (2.2 mL, 0.5 M) was slowly added to a solution of **12b** (494.0 mg, 1.11 mmol) in MeCN (2.2 mL) at room temperature. After being stirred for 5 min, the reaction mixture was diluted with H_2O (20 mL) and extracted with EtOAc (10 mL x3). The combined organic layer was dried over Na_2SO_4, filtered and concentrated. The residue was purified with flash column chromatography (silica gel; Hex/EtOAc = 10/1) to afford to **SI5b** (435.0 mg, 94% yield) as a yellow solid.
$[\alpha]_D^{26.6}$: +19.3 ° (c = 0.1, $CHCl_3$).
m.p.: 172.7-172.9 °C.
IR (diamond prism)νcm^{-1}: 3969, 3931, 1739, 1666, 1519, 1365, 1284, 1230, 1153, 1095, 1022, 941, 894, 848, 755, 690.
^{1}H NMR (500 MHz, $CDCl_3$)δ(ppm): 8.44 (d, J = 1.7 Hz, 1H), 8.22 (dd, J = 8.0, 1.7 Hz, 1H), 8.19 (d, J = 8.0 Hz, 1H), 7.10 (d, J = 8.0 Hz, 1H), 6.90 (d, J = 1.7 Hz, 1H), 4.81 (ddd, J = 8.9, 8.6, 8.0 Hz, 1H), 3.88 (dd, J = 8.6, 2.9 Hz, 1H), 3.69 (dd, J = 8.9, 2.9 Hz, 1H), 2.22 (d, J = 1.7 Hz, 3H), 1.50 (s, 9H), 1.17 (s, 9H).
^{13}C NMR (125 MHz, $CDCl_3$)δ(ppm): 184.9, 184.1, 169.3, 165.2, 148.5, 138.9, 135.9, 133.8, 132.5, 132.3, 127.1, 124.5, 82.3, 73.3, 62.1, 53.9, 28.1, 27.4, 16.5.
HRMS (EI) m/z 438.1890 [M+Na]+, calcd 438.1893 for $C_{23}H_{29}NO_6Na$.

<u>(6-methyl-5,8-dioxo-5,8-dihydronaphthalene-2-carbonyl)-L-serine (**13b**):</u>

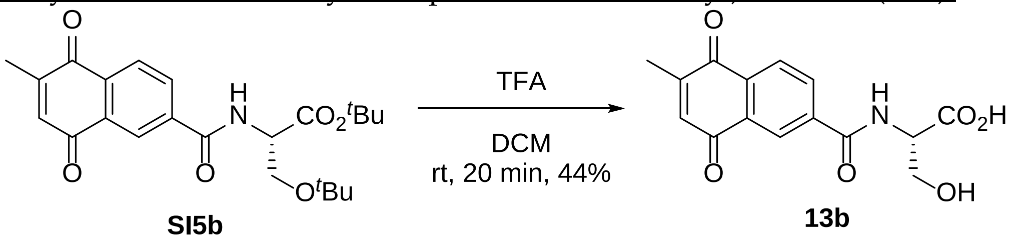

SI5b (100.0 mg, 0.24 mmol) was dissolved in 50% TFA/DCM (30 mL, 0.01 M), and the reaction mixture was stirred at room temperature. After being stirred for 20 min, the reaction mixture was concentrated. The residue was purified with column chromatography (silica gel; $CHCl_3$/MeOH = 10/1) to afford **13b** (32.0 mg, 44% yield) as a light brownish yellow solid.
$[\alpha]_D^{27.1}$: +26.3 ° (c = 0.1, H_2O).
m.p.: 166.6-166.8 °C.
IR (diamond prism)νcm^{-1}: 3262, 1739, 1646, 1353, 1280, 1218, 1157, 1110, 1060, 944, 879, 755, 690.
^{1}H NMR (500 MHz, DMSO-d_6)δ(ppm): 8.93 (d, J = 8.0 Hz, 1H), 8.47 (d, J = 1.7 Hz, 1H), 8.29 (dd, J = 8.0, 1.7 Hz, 1H), 8.10 (d, J = 8.0 Hz, 1H), 7.06 (d, J = 1.7 Hz, 1H), 4.52 (td, J = 8.0, 5.2 Hz, 1H), 3.82 (d, J = 5.2 Hz, 1H), 2.14 (d, J = 1.3 Hz, 3H).
^{13}C NMR (125 MHz, DMSO-d_6)δ(ppm): 184.6, 184.2, 171.6, 165.0, 148.4, 135.5, 133.5, 132.6, 131.8, 126.4, 124.6, 61.0, 55.9, 16.0.
HRMS (EI) m/z 302.0666 [M-H]-, calcd 302.0664 for $C_{15}H_{12}NO_6$.

Supplementary Figures

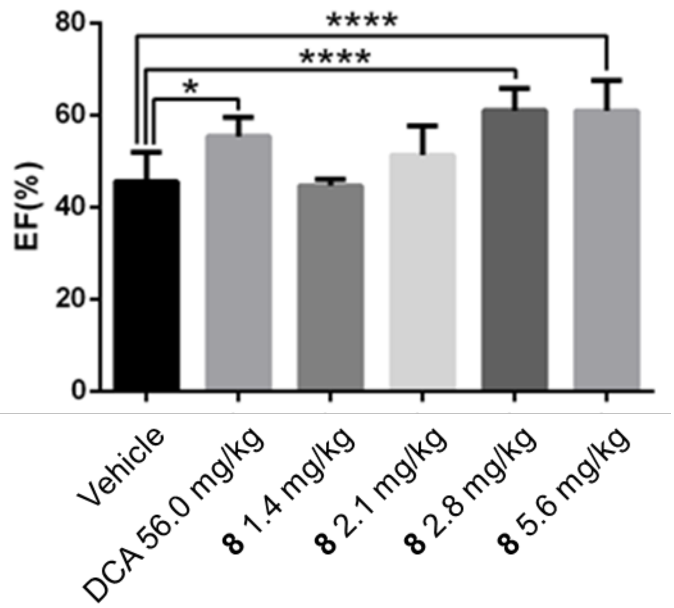

Supplementary Figure S1. Comparison of ejection fraction after administration of **8** by various doses (vehicle or 1.4, 2.1, 2.8, or 5.6 mg/kg). **8** were administered once a day between 4 and 5 weeks after TAC. * p<0.05, ****p<0.0001, Data were presented as Mean ± S.E., TAC model mice group: n=25, other groups: n=5

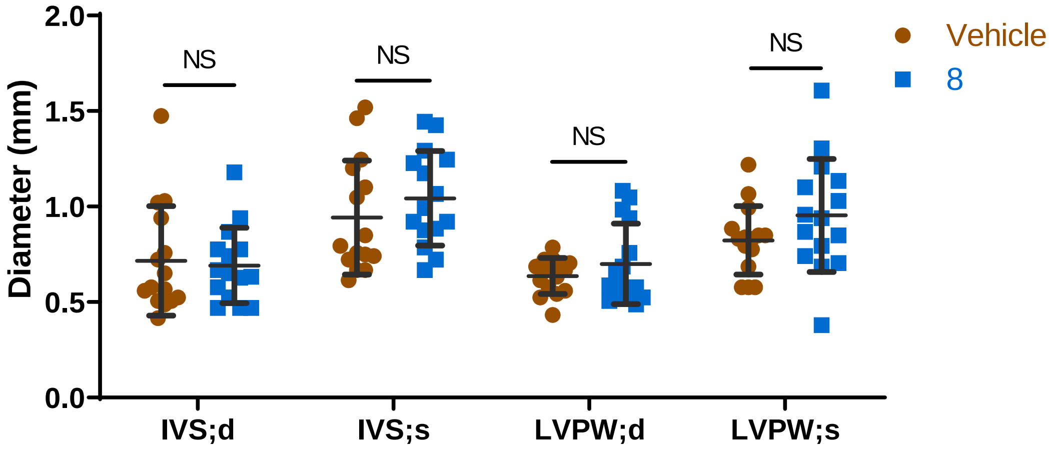

Supplementary Figure S2. Comparison of interventricular septum thickness at end-diastole and at end-systole (IVS;d and IVS;s) and left ventricle posterior wall thickness at end-diastole and at end-systole (LVPW;d and LVPW;s) between vehicle-injected TAC mice and **8**-injected TAC mice. Each echocardiographic parameter was measured after 4 weeks of an observation period and 1 week of vehicle or **8** treatment. NS = not significant according to the Student's t-test.

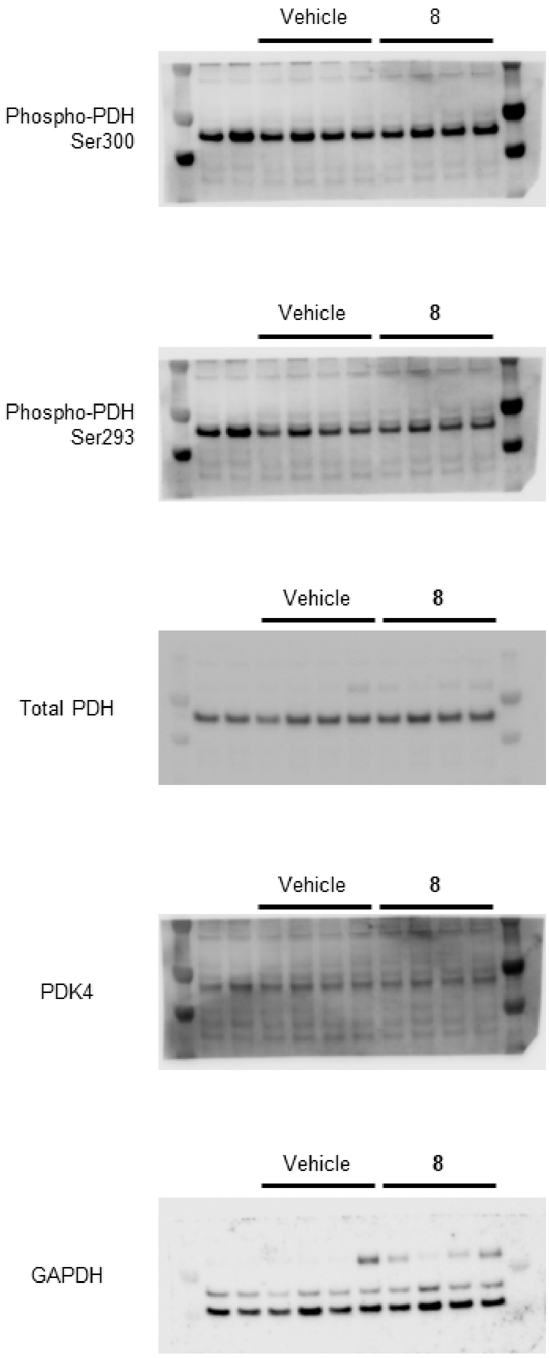

Supplementary Figure S3. Uncropped western blot membranes. The images show the original western blot membranes where the bands in Figure 4e are from.

References

1. Maloney, D. J.; Hecht, S. M., A stereocontrolled synthesis of delta-trans-tocotrienoloic acid. *Org Lett* **2005,** 7, (19), 4297–300.
2. Nyland, R. L., II; Luo, M.; Kelley, M. R.; Borch, R. F., Design and Synthesis of Novel Quinone Inhibitors Targeted to the Redox Function of Apurinic/Apyrimidinic Endonuclease 1/Redox

Enhancing Factor-1 (Ape1/Ref-1). *Journal of Medicinal Chemistry* **2010,** 53, (3), 1200–1210.
3. Joshi, P. L.; Dhekne, V. V.; Rao, A. S., Synthesis of 6-acetyl-1,4-dimethoxy-2-methylnaphthalene. *Indian J Chem B* **1983,** 22, (1), 23–26.
